# Acute respiratory infection symptoms and COVID-19 testing behaviour: results based on South Australian health surveys

**DOI:** 10.1186/s12889-021-12359-3

**Published:** 2021-12-20

**Authors:** S. Joshi, K. D’Onise, R. Nolan, S. Davis, K. Glass, K. Lokuge

**Affiliations:** 1 Epidemiology Branch, Prevention and Population Health, Wellbeing SA, Government of South Australia, Level 9, The Conservatory, Rundle Mall, PO BOX 388, Adelaide, SA 5000 Australia; 2 Humanitarian Health Research Initiative, Research School of Population Health, Australian National University, 62A Mills Road, Canberra, ACT 2601 Australia; 3 National Centre for Epidemiology and Population Health, Australian National University, 62A Mills Road, ACT 2601 Canberra, Australia

**Keywords:** ARI, COVID-19, Coronavirus testing, Population survey, Syndromic surveillance

## Abstract

**Background:**

Effective syndromic surveillance alongside COVID-19 testing behaviours in the population including in higher risk and hard to reach subgroups is vital to detect re-emergence of COVID-19 transmission in the community. The aim of this paper was to identify the prevalence of acute respiratory infection symptoms and coronavirus testing behaviour among South Australians using data from a population based survey.

**Methods:**

We used cross-sectional data from the 2020 state-wide population level health survey on 6857 respondents aged 18 years and above. Descriptive statistics were used to explore the risk factors and multivariable logistic regression models were used to assess the factors associated with the acute respiratory infection symptoms and coronavirus testing behaviour after adjusting for gender, age, household size, household income, Aboriginal and/or Torres Strait Islander status, SEIFA, Country of birth, number of chronic diseases, wellbeing, psychological distress, and mental health.

**Results:**

We found that 19.3% of respondents reported having symptoms of acute respiratory infection and the most commonly reported symptoms were a runny nose (11.2%), coughing (9.9%) and sore throat (6.2%). Fever and cough were reported by 0.8% of participants. Of the symptomatic respondents, 32.6% reported seeking health advice from a nurse, doctor or healthcare provider. Around 18% (*n* = 130) of symptomatic respondents had sought testing and a further 4.3% (*n* = 31) reported they intended to get tested. The regression results suggest that older age, larger household size, a higher number of chronic disease, mental health condition, poor wellbeing, and psychological distress were associated with higher odds of ARI symptoms. Higher household income was associated with lower odds of being tested or intending to be tested for coronavirus after adjusting for other explanatory variables.

**Conclusions:**

There were relatively high rates of self-reported acute respiratory infection during a period of very low COVID-19 prevalence and low rate of coronavirus testing among symptomatic respondents. Ongoing monitoring of testing uptake, including in higher-risk groups, and possible interventions to improve testing uptake is key to early detection of disease.

## Background

The current Coronavirus Disease 2019 (COVID-19) pandemic is presenting challenges to health and welfare systems worldwide. Prior to effective vaccine or pharmaceutical treatments, options for control are limited to traditional public health actions: primarily individual measures of early identification and isolation or quarantine of cases and controls (respectively) [1] and societal wide measures including social distancing and government mandated stay-at-home orders [2]. Even once vaccines and other therapeutics become available, public health systems will continue to be critical to effective identification and management of disease.

Surveillance is a core function of a public health system and is imperative to the control of COVID-19 [3]. Australia’s surveillance strategy is based on testing those with symptomatic disease in the community [4–6], and this has underpinned an effective public health response to COVID-19 to date. However, the effectiveness of this strategy relies on high uptake of testing in the community. Even with overall good availability of testing and health messaging on the public health importance of being tested, uptake may not be optimal. Various factors may contribute to non-optimal uptake, ranging from the generally mild illness caused by COVID-19 amongst young people [7], to social factors including lack of access to health care, inability to isolate and stay away from work whilst awaiting test results [8], or to limited access to mainstream health promotion messaging [9] due to language barriers. Such factors have the potential to have greater impact on high-risk populations, running the risk of undetected amplification within these groups [10, 11]. This undetected amplification occurred in Australia; when COVID-19 first emerged in Australia in March and April 2020, transmission was suppressed through widespread stay at home orders. However, re-emergence, initiated by failures in border quarantine measures, was sustained and amplified through generations of undetected transmission in hard-to-reach populations. This resulted in a large outbreak in the state of Victoria, which has caused more than 18,000 cases and 700 deaths as of 13 January 2021 [12].

Effective surveillance, including in higher-risk and hard-to-reach subgroups, is therefore crucial to detecting and controlling COVID-19, both when transmission is high and when community transmission has largely been suppressed, so that re-emergence is detected as early as possible. However, testing data alone has limitations in determining surveillance and testing coverage in the community, as total numbers of tests do not indicate the proportion of symptomatic people presenting for testing. Sentinel surveillance systems based on volunteers who self-report illness are also limited, as those who participate in such systems are likely to differ from the general community.

To determine testing coverage in those with symptoms, community-based representative data is therefore essential. Such information enables public health authorities to identify groups at risk of infection and groups unlikely to seek testing. This paper reports on a cross-sectional survey of adults in South Australia that can be weighted to give population-level estimates of acute respiratory infection (ARI) and testing uptake, including in groups potentially at higher risk of COVID-19 infection and those less likely to participate in testing.

## Methods

### Study design and data sources

This study used a cross-sectional survey design of South Australian adults aged 18 years and over.

Two surveys were used to collect data: the South Australian Population Health Survey (SAPHS) and the Population Health Survey Module System (PHSMS). The SAPHS is an ongoing state-wide population level survey which has been monitoring the health and wellbeing of South Australians since 2018. Data are collected in an ongoing way. The PHSMS utilises the same methodology and sampling frame (the South Australian population) as the SAPHS, however is a discrete survey of *n* = 3000 respondents, different to those who completed the SAPHS. Identical questions were asked across both surveys, and therefore the data collected have been combined.

### Sampling

A probability based, dual-frame sample of South Australian adults was selected using random digit dialling. Standard de-duplication and weighting procedures for the dual over-lapping frames were administered. As both surveys are population level surveys, the only inclusion criteria were that respondents be residents of South Australia with access to a telephone. The data collector was able to conduct interviews in selected languages other than English. Selected persons were non-replaceable, such that if the selected person was not available, interviews were not conducted with alternative household members (landline) or a person who was not the owner of the phone (mobile). There were no other exclusion criteria for the survey, although we additionally excluded children under 18 from our analysis.

### Data collection

Data were primarily collected using computer assisted telephone interviewing (CATI). To maximise participation, respondents were also offered the option of completing the survey on-line (CAWI, computer-assisted web interview) by receiving a unique hyperlink. The data presented in this paper were collected from 2 April to 28 June 2020 (SAPHS) and 14 May to 28 June 2020 (PHSMS).

In response to the COVID-19 pandemic, questions were included in both surveys to monitor symptoms of ARI in the adult population. Respondents were asked if they currently (or within the last week) had any symptoms of ARI consistent with coronavirus (fever, cough, sore throat, runny nose, shortness of breath, or a change of sense of taste or smell). Subsequent data collected in SAPHS from October 2020 suggests that 38% of symptomatic respondents have acute symptoms. All symptomatic respondents were also asked if they had sought health advice.

Finally, a subset of the sample was asked a question about testing behaviour. From 29 May 2020 (PHSMS) and 01 June 2020 (SAPHS), symptomatic respondents were asked if their symptoms were current on the day of the interview, and whether or not they had presented, or intended to present for COVID-19 testing. A sensitivity analysis showed no difference between those who had presented and those who intended to present for COVID-19 testing, and therefore these groups have been combined for analysis.

Data are presented by various demographic and population sub-groups. Socio-Economic Index For Areas (SEIFA) quintiles are presented according to the definition from the Australian Bureau of Statistics [13] using the respondent’s postcode and suburb. The respondent’s country of birth has been divided into two broad categories: English speaking countries and non-English speaking countries. Respondents who were born in Australia, the United Kingdom, Ireland, the United States of America, New Zealand, Canada, and South Africa are included in the English-speaking background category. All other respondents are included in the non-English speaking background category [14]. Health outcomes presented in the inferential analyses are self-reported according to whether the respondent had even been told by a doctor or nurse they had the condition. Psychological distress was measured using the Kessler Psychological Distress Scale (K10) [15]. Subjective wellbeing was measured using the United Kingdom Office for National Statistics 4-item questionnaire [16].

### Analysis

Data preparation and analysis were completed using SPSS 24 software. Excel 2010 was used to collate tables. Weighted data were used for the descriptive analysis and unweighted data were used for regression analysis.

Descriptive statistics were used to explore the associations between demographic and health outcome variables and ARI symptoms and testing behaviour. Counts and percentages were used to describe categorical data. Chi-square tests were applied to test the associations between the exposure variables and ARI symptoms and testing behaviour. The weighted proportions of people who respond to each category of the attribute are presented in the tables along with the 95% confidence interval. Raking [17] was used to weight respondents incorporating various population characteristics (gender, age, area of residence, country of birth, dwelling status, marital status, education level, employment status, household size) to more closely reflect the South Australian population using benchmarks derived from the June 2016 ABS Census data. The weighting of data can result in rounding discrepancies or totals not adding. Non-relevant responses such as ‘don’t know’ and ‘refused’ have not been included in the analysis except where stated.

Multiple logistic regression analyses using unweighted data was used to investigate the multiple factors related to ARI symptoms and testing behaviour. Potentially explanatory variables on the basis of known increased risk for respiratory viral transmission and the univariate analyses above such as gender, age, household size, household income, Aboriginal and/or Torres Strait Islanders status, SEIFA, Country of birth, number of chronic diseases, wellbeing, psychological distress, and mental health were considered in the multivariable analyses. A sub model including smoking status and private health insurance was performed using the SAPHS dataset only as these questions were not asked in the PHSMS survey.

## Results

### Demographic profile of survey respondents

The weighted and unweighted sample of the SAPHS and PHSMS 2020 surveys in this study are presented in (Table 1). The population groups underrepresented by the survey include adults aged 18–29 years and people from non-English speaking backgrounds, while there was overrepresentation of adults 50 to 69 years, people with a degree or higher education level, people who earn over $150,000, and people who are retired.

**Table 1:** Weighted and unweighted demographic sample of SAPHS and PHSMS, April–June 2020

	Weighted (***n*** = 6857)		Unweighted (***n*** = 7086)	
	n	% (95% CI)	n	% (95% CI)
All	**6857**	**100.0**	**7086**	**100.0**
Gender^a^				
Male	3319	48.4 (47.2–49.6)	2936	41.4 (40.3–42.6)
Female	3537	51.6 (50.4–52.8)	4149	58.6 (57.4–59.7)
Location				
Metropolitan	4964	72.4 (71.3–73.5)	4884	68.9 (67.9–70.0)
Rural	1890	27.6 (26.5–28.6)	2200	31.1 (30.0–32.1)
Age (years)				
18 to 29	1356	19.8 (18.8–20.7)	268	3.8 (3.4–4.2)
30 to 49	1809	26.4 (25.3–27.4)	1480	20.9 (20.0–21.8)
50 to 69	2057	30.0 (28.9–31.1)	3548	50.1 (48.9–51.2)
70 and over	1634	23.8 (22.8–24.8)	1790	25.3 (24.3–26.3)
SEIFA^b^				
Lowest	1245	18.2 (17.3–19.1)	1111	15.7 (14.9–16.6)
Low	1544	22.6 (21.6–23.6)	1539	21.7 (20.8–22.7)
Middle	1526	22.3 (21.3–23.3)	1494	21.1 (20.2–22.1)
High	1210	17.7 (16.8–18.6)	1294	18.3 (17.4–19.2)
Highest	1320	19.3 (18.4–20.2)	1641	23.2 (22.2–24.2)
Highest education level				
No school to secondary	2774	40.5 (39.3–41.6)	2578	36.4 (35.3–37.5)
TAFE, trade, certificate	1369	20.0 (19.0–20.9)	1113	15.7 (14.9–16.6)
Diploma, advanced diploma	947	13.8 (13.0–14.6)	920	13.0 (12.2–13.8)
Degree or higher	1731	25.3 (24.2–26.3)	2446	34.5 (33.4–35.6)
Not stated	36	0.5 (0.4–0.7)	29	0.4 (0.3–0.6)
Household Income				
Up to $20,000	536	7.8 (7.2–8.5)	512	7.2 (6.6–7.8)
$20,001 - $40,000	1118	16.3 (15.4–17.2)	1114	15.7 (14.9–16.6)
$40,001 - $60,000	812	11.8 (11.1–12.6)	889	12.5 (11.8–13.3)
$60,001 - $80,000	780	11.4 (10.6–12.1)	732	10.3 (9.6–11.1)
$80,001 - $100,000	575	8.4 (7.7–9.1)	625	8.8 (8.2–9.5)
$100,001 - $150,000	760	11.1 (10.4–11.8)	842	11.9 (11.1–12.7)
More than $150,000	575	8.4 (7.7–9.1)	797	11.2 (10.5–12.0)
Not stated	1701	24.8 (23.8–25.8)	1575	22.2 (21.3–23.2)
Country of birth				
English speaking background	5711	83.3 (82.4–84.2)	6354	89.7 (88.9–90.4)
Non-English speaking background	1114	16.2 (15.4–17.1)	708	10.0 (9.3–10.7)
Not stated/defined	33	0.5 (0.3–0.7)	24	0.3 (0.2–0.5)
Language spoken at home				
English	6274	91.5 (90.8–92.1)	6778	95.7 (95.2–96.1)
Other	575	8.4 (7.7–9.1)	301	4.2 (3.8–4.7)
Not stated	7	0.1 (0.0–0.2)	7	0.1 (0.0–0.2)
Aboriginal and/or Torres Strait Islander Status				
Yes	136	2.0 (1.7–2.3)	93	1.3 (1.1–1.6)
No	6675	97.3 (96.9–97.7)	6963	98.3 (97.9–98.5)
Not stated	46	0.7 (0.5–0.9)	30	0.4 (0.3–0.6)
Work status				
Employed^	3425	50.0 (48.8–51.1)	3510	49.5 (48.4–50.7)
Unemployed	273	4.0 (3.5–4.5)	372	5.2 (4.7–5.8)
Student	335	4.9 (4.4–5.4)	80	1.1 (0.9–1.4)
Retired	1989	29.0 (27.9–30.1)	2506	35.4 (34.3–36.5)
Other^^	796	11.6 (10.9–12.4)	591	8.3 (7.7–9.0)
Not stated	39	0.6 (0.4–0.8)	27	0.4 (0.3–0.5)
Household size				
1	2108	30.7 (29.7–31.8)	1809	25.5 (24.5–26.6)
2	2605	38.0 (36.8–39.1)	3126	44.1 (43.0–45.3)
3	892	13.0 (12.2–13.8)	848	12.0 (11.2–12.7)
4	675	9.8 (9.2–10.6)	775	10.9 (10.2–11.7)
5 or more	513	7.5 (6.9–8.1)	481	6.8 (6.2–7.4)
Not stated	65	0.9 (0.7–1.2)	47	0.7 (0.5–0.9)

*Note*: the weighting of the data can result in rounding discrepancies or totals not adding. *CI:* Confidence Interval

^a^Not shown 1 respondent reporting gender as ‘other’. ^b^SEIFA: Socio-Economic Index For Areas

^Employed included full time employed, part time employed and casual employment

^^Other category includes respondents engaged in home duties, Unable to work, Carer, Volunteers, and any other responses

Of the 6857 respondents included in this study, 48.4% were male and 51.6% were female, most resided in metropolitan areas (72.4%), were from an English-speaking background (83.3%), spoke English at home (91.5%), had less than four people living in their household (81.7%), and were employed (50.0%).

### Descriptive analysis results

Across both surveys, 6833 respondents were asked if they had any symptoms of ARI, with the majority (80.7%) not reporting any symptoms. Of those who reported symptoms (*n* = 1318, 19.3%) the most commonly reported symptoms were a runny nose (11.2%), coughing (9.9%), sore throat (6.2%), and shortness of breath (6.0%). Less than one percent % (0.8%) of respondents reported having fever and cough in the past week. Weekly estimates of symptomatic respondents ranged from 9.9 to 22.7% over the 13-week study period. Symptomatic respondents were then asked if they had sought health advice from a nurse, doctor, or other health care provider. Of the 1312 people who responded to this question, 32.6% reported seeking health advice from a nurse, doctor or other healthcare provider.

The proportions of respondents reporting ARI symptoms by selected demographic and health outcome measures are presented in (Table 2). Females were more likely to report ARI symptoms than males. The likelihood of reporting ARI symptoms tended to be higher in lower household income groups and in Aboriginal and/or Torres Strait Islander respondents compared with non-Aboriginal and/or Torres Strait Islander respondents. The symptom report proportion increased with increasing household members, although this was not statistically significant.

**Table 2:** Proportion of respondents reporting ARI symptoms in the prior week, by key demographic and health outcome variables, (SAPHS & PHSMS, April – June 2020, **N**^†^ **= 6833**)

	n/N	% (95% CI)	***P***-value
**All**	**1318/6833**	**19.3 (18.4–20.2)**	
**Gender**^a^			
Male	559/3311	16.9 (15.6–18.2)	< 0.001
Female	758/3522	21.5 (20.2–22.9)	
**Age**			
18–29	272/1347	20.2 (18.1–22.4)	0.108
30–49	374/1805	20.8 (18.9–22.6)	
50–69	381/2050	18.6 (16.9–20.3)	
70 and over	290/1631	17.8 (16.0–19.7)	
**Location**			
Metropolitan	960/4949	19.4 (18.3–20.5)	0.695
Rural	357/1881	19.0 (17.3–20.8)	
**SEIFA**^b^			
Lowest	267/1236	21.6 (19.4–24.0)	0.071
Low	313/1539	20.4 (18.4–22.4)	
Middle	281/1520	18.5 (16.6–20.5)	
High	224/1206	18.6 (16.5–20.8)	
Highest	233/1319	17.7 (15.7–19.8)	
**Education**			
No school to secondary	492/2761	17.8 (16.4–19.3)	< 0.001
TAFE, trade, certificate	324/1367	23.7 (21.5–26.0)	
Diploma, advanced diploma	181/945	19.2 (16.7–21.8)	
Degree or higher	319/1726	18.5 (16.7–20.4)	
**Household Income**			
Up to $20,000	125/535	23.3 (19.9–27.1)	< 0.001
$20,001 - $40,000	263/1116	23.6 (21.1–26.1)	
$40,001 - $60,000	182/812	22.4 (19.6–25.4)	
$60,001 - $80,000	158/779	20.2 (17.6–23.2)	
$80,001 - $100,000	91/573	15.9 (13.1–19.0)	
$100,001 - $150,000	140/758	18.5 (15.8–21.3)	
More than $150,000	93/574	16.3 (13.4–19.4)	
Not stated	267/1685	15.8 (14.2–17.6)	
**Country of birth**			
English speaking background	1114/5699	19.5 (18.5–20.6)	0.276
Non-English speaking background	200/1104	18.2 (15.9–20.5)	
**Aboriginal status**			
Aboriginal and/or Torres Strait Islanders	40/136	29.2 (22.2–37.4)	0.003
Non-Aboriginal and/or Torres Strait Islanders	1272/6652	19.1 (18.2–20.1)	
**Household size**			
1	369/2105	17.5 (16.0–19.2)	0.004
2	476/2597	18.3 (16.9–19.9)	
3	179/891	20.1 (17.6–22.8)	
4	158/673	23.5 (20.4–26.8)	
5 or more	108/502	21.6 (18.1–25.3)	
**Chronic conditions**^c^			
No chronic condition	463/3029	15.3 (14.0–16.6)	< 0.001
1 chronic condition	423/2042	20.7 (19.0–22.5)	
2 chronic condition	206/1057	19.5 (17.2–22.0)	
3+ chronic condition	225/704	31.9 (28.6–35.5)	
**Overall subjective wellbeing**			
Good wellbeing	250/2449	10.2 (9.1–11.5)	< 0.001
Neutral	518/2438	21.2 (19.7–22.9)	
Poor wellbeing	506/1792	28.2 (26.2–30.4)	
**Mental health conditions**^d^			
Yes	519/1727	30.1 (27.9–32.2)	< 0.001
No	784/5059	15.5 (14.5–16.5)	
**Psychological distress (K10)**			
Yes	472/1279	36.9 (34.3–39.6)	< 0.001
No	827/5467	15.1 (14.2–16.1)	

*Note*: The weighting of data can result in rounding discrepancies or totals not adding. *CI:* Confidence Interval

^a^Respondents reporting gender as other (*n* = 1) excluded. ^b^SEIFA: Socio-Economic Index For Areas.

^c^Chronic health condition includes diabetes, asthma, COPD, CVD, arthritis, osteoporosis, and cancer

^d^Mental health condition includes anxiety, depression, stress related problem, and other mental health condition

^†^Don’t know/refused responses excluded

Respondents who had at least three chronic conditions were more likely to report ARI symptoms than who did not have these conditions. Respondents who had poor overall wellbeing, mental health conditions (including anxiety, depression, stress related problem, and other mental health conditions), and psychological distress were also more likely to report ARI (Table 2).

A subset of symptomatic respondents (*n* = 722/3544) were further asked if their symptoms were present on the day of interview and whether they had presented, or intend to, for COVID-19 testing. Around half (*n* = 365) of symptomatic respondents reported ARI symptoms on the day of the interview, representing 10.3% of all respondents. Most symptomatic respondents (*n* = 562, 78%) had not presented for tested nor planned to do so. Around 18% (*n* = 130) had sought testing and a further 4.3% (*n* = 31) said they intended to get tested.

The proportions of symptomatic respondents reporting being tested or intending to be tested for COVID-19 by selected demographic measures and health outcome measures are presented in (Table 3). The likelihood of reporting COVID-19 testing was significantly higher in the highest SEIFA group and those with an English speaking background. The proportion of respondents reporting COVID-19 testing was lower among respondents who had household income of $100,001- $150,000 and more than $150,000.

**Table 3:** Proportion of symptomatic respondents reporting being tested or intending to be tested, by key demographic and health outcome variables, (SAPHS & PHSMS, May–June 2020, ***N***^†^ **= 722**)

	n/N	% (95% CI)	***P***-value
**All**	**161/722**	**22.2 (19.4–25.4)**	
**Gender**^a^			
Male	60/299	20.2 (15.8–24.9)	0.255
Female	100/423	23.7 (19.8–27.9)	
**Age**			
18–29	44/165	26.8 (20.4–33.8)	0.110
30–49	45/185	24.4 (18.6–30.9)	
50–69	42/194	21.7 (16.3–27.8)	
70 and over	29/178	16.2 (11.4–22.2)	
**Location**			
Metropolitan	109/505	21.5 (18.2–25.3)	0.501
Rural	52/218	23.8 (18.6–29.8)	
**SEIFA**^b^			
Lowest	23/134	17.1 (11.5–24.2)	0.006
Low	47/206	22.7 (17.5–28.9)	
Middle	33/124	26.7 (19.4–34.9)	
High	17/126	13.6 (8.4–20.3)	
Highest	41/133	30.6 (23.5–39.0)	
**Education**			
No school to secondary	55/269	20.5 (16.0–25.6)	0.017
TAFE, trade, certificate	37/166	22.4 (16.5–29.1)	
Diploma, advanced diploma	34/100	34.3 (25.3–43.6)	
Degree or higher	34/186	18.2 (13.2–24.3)	
**Household Income**			
Up to $20,000	10/55	18.4 (9.7–29.9)	0.039
$20,001 - $40,000	42/154	27.4 (20.7–34.7)	
$40,001 - $60,000	25/90	27.8 (19.3–37.6)	
$60,001 - $80,000	18/67	26.2 (17.4–38.3)	
$80,001 - $100,000	15/52	28.6 (17.9–42.1)	
$100,001 - $150,000	11/84	13.1 (7.2–21.5)	
More than $150,000	6/53	10.8 (4.9–21.9)	
Not stated	34/167	20.3 (14.8–26.9)	
**Country of birth**			
English speaking background	153/634	24.1 (20.9–27.6)	0.001
Non-English speaking background	8/88	8.8 (4.4–16.4)	
**Aboriginal status**			
Aboriginal and/or Torres Strait Islanders	7/18	37.7 (19.4–61.7)	0.088
Non-Aboriginal and/or Torres Strait Islanders	154/702	21.9 (19.0–25.1)	
**Household size**			
1	42/196	21.6 (16.1–27.6)	0.809
2	54/224	24.2 (18.9–30.0)	
3	29/121	24.0 (17.0–32.1)	
4	20/107	18.4 (12.2–26.9)	
5 or more	15/72	20.6 (12.7–31.2)	
**Chronic conditions**^c^			
No chronic condition	44/252	17.3 (13.2–22.5)	< 0.001
1 chronic condition	42/238	17.7 (13.2–22.9)	
2 chronic condition	31/110	28.2 (20.4–37.1)	
3+ chronic condition	44/122	35.8 (28.0–44.8)	
**Overall subjective wellbeing**			
Good wellbeing	20/140	14.4 (9.2–20.8)	0.008
Neutral	64/289	22.0 (17.7–27.2)	
Poor wellbeing	76/273	27.9 (22.8–33.4)	
**Mental health condition**^d^			
Yes	88/299	29.3 (24.5–34.8)	< 0.001
No	73/419	17.4 (14.0–21.3)	
**Psychological distress (K10)**			
Yes	75/225	33.5 (27.4–39.7)	< 0.001
No	82/484	17.0 (13.8–20.5)	

*Note*: The weighting of data can result in rounding discrepancies or totals not adding. *CI:* Confidence Interval

^a^Respondents reporting gender as other (*n* = 1) excluded. ^b^SEIFA: Socio-Economic Index for Areas.

^c^Chronic health condition includes diabetes, asthma, COPD, CVD, arthritis, osteoporosis, and cancer

^d^Mental health condition includes anxiety, depression, stress related problem, and other mental health condition

^†^Don’t know/refused responses excluded

Symptomatic respondents who had and at least three chronic conditions were more likely to report being tested or the intent to be tested for COVID-19. Respondents who had poor overall wellbeing, mental health conditions (includes anxiety, depression, stress related problem, and other mental health conditions), and psychological distress were also more likely to report for being tested for COVID-19 (Table 3).

### Regression analysis results

The multivariate logistic regression analysis in Table 4 provides odds ratios (OR) for respondents reporting ARI symptoms compared to those not having ARI symptoms after adjusting for other explanatory variables in the model. The regression results suggest that older age (OR 0.99, 95% CI 0.98–0.99) larger household size (OR 1.06, 95% CI 1.01–1.12), and a higher number of chronic health conditions (OR 1.31, 95% CI 1.22–1.40) were associated with higher odds of ARI symptoms. Respondents who had a mental health condition (OR 1.27, 95% CI 1.06–1.53), poor wellbeing (OR 1.83, 95% CI 1.54–2.17) and those with psychological distress (OR 1.69, 95% CI 1.37–2.07) had higher odds of ARI symptoms. Aboriginal and/or Torres Strait Islander participants had an odds of reporting ARI of 1.46 (95% CI 0.86–2.49) compared to non-Aboriginal and/or Torres Strait Islander participants, estimated on a small number of participants and so with low precision.

**Table 4:** Multivariate logistic regression for key demographic factors and health outcome associated with respondents (18+ years) reporting ARI symptoms (SAPHS & PHSMS, April – June 2020, ***N***† **= 7063**)

	Odds Ratio	95% CI
Females	Reference	
Males	0.96	0.83–1.12
Age (continuous)	0.99^*^	0.98–0.99
Household size (continuous)	1.06^*^	1.01–1.12
SEIFA		
Lowest	Reference	
Low	1.05	0.83–1.33
Middle	1.04	0.82–1.32
High	0.89	0.69–1.15
Highest	0.99	0.77–1.27
Household income		
Up to $20,000	Reference	
$20,001–$40,000	0.84	0.63–1.10
$40,001–$60,000	0.98	0.74–1.32
$60,0001–$80,000	0.93	0.68–1.27
$80,000–$100,000	0.87	0.62–1.20
$100,001–$150,000	0.83	0.60–1.14
More than $150,000	0.78	0.56–1.09
Non-Aboriginal and/or Torres Strait Islanders	Reference	
Aboriginal and/or Torres Strait Islanders	1.46	0.86–2.49
Country of Birth		
English speaking background	Reference	
Non-English speaking background	0.95	0.73–1.23
Number of chronic conditions^a^ (continuous)	1.31^*^	1.22–1.40
Good subjective overall wellbeing	Reference	
Poor wellbeing or neutral wellbeing	1.83^*^	1.54–2.17
No mental health condition	Reference	
Any mental health condition^b^	1.27^*^	1.06–1.53
No psychological distress	Reference	
Psychological distress	1.69^*^	1.37–2.07
Likelihood ratio test (−2 LogL) = **4660.2**		

*Note*: Unweighted data used for regression analysis. *CI:* Confidence Interval. †Don’t know/Refused excluded for ARI symptoms

Respondents reporting gender as other (*n* = 1) excluded. SEIFA: Socio-Economic Index For Areas

^*^Denotes statistical significance (*p* < 0.05) compared to the reference category

^a^Chronic health condition includes diabetes, asthma, COPD, CVD, arthritis, osteoporosis, and cancer

^b^includes anxiety, depression, stress, or any other mental health conditions in the previous 12 months

The multivariate logistic regression analysis in Table 5 provides odds ratios (OR) for respondents reporting being tested or intending to be tested for COVID-19 compared to those not tested for COVID-19 after adjusting for other explanatory variables in the model. The regression results suggest that respondents who had household income of $100,001- $150,000 (OR 0.40, 95% CI 0.16–0.98) and more than $150,000 (OR 0.32, 95% CI 0.12–0.86) were associated with lower odds of COVID testing. Aboriginal and/or Torres Strait Islander participants had an odds of reporting COVID-19 testing of 2.42 (95% CI 0.59–9.88) compared to non-Aboriginal and/or Torres Strait Islander participants, estimated on a small number of participants and so with low precision.

**Table 5:** Multivariate logistic regression for key demographic factors and health outcome associated with respondents (18+ years) reporting being tested or intending to be tested for COVID-19 (SAPHS & PHSMS, May – June 2020, **N† = 656**)

	Odds Ratio	95% CI
Females	Reference	
Males	1.16	0.72–1.86
Age (continuous)	0.99	0.97–1.01
Household size (continuous)	1.07	0.91–1.26
SEIFA		
Lowest	Reference	
Low	1.21	0.59–2.47
Middle	1.29	0.63–2.66
High	0.86	0.39–1.91
Highest	1.48	0.71–3.09
Household Income		
Up to $20,000	Reference	
$20,001–$40,000	0.57	0.27–1.22
$40,001–$60,000	0.53	0.23–1.20
$60,0001–$80,000	0.79	0.35–1.82
$80,000–$100,000	0.75	0.31–1.86
$100,001–$150,000	0.40^*^	0.16–0.98
More than $150,000	0.32^*^	0.12–0.86
Non-Aboriginal and/or Torres Strait Islanders	Reference	
Aboriginal and/or Torres Strait Islanders	2.42	0.59–9.88
Country of Birth		
English speaking background	Reference	
Non-English speaking background	0.69	0.30–1.57
Number of chronic conditions^a^ (continuous)	0.94	0.77–1.16
Good subjective overall wellbeing	Reference	
Poor wellbeing or neutral wellbeing	1.04	0.60–1.79
No mental health condition	Reference	
Any mental health condition^b^	1.00	0.58–1.73
No psychological distress	Reference	
Psychological distress	1.26	0.71–2.26
Likelihood ratio test (−2 LogL) = **513.7**		

*Note*: Unweighted data used for regression analysis. *CI:* Confidence Interval. †Don’t know/Refused excluded for COVID-19 testing

Respondents reporting gender as other (*n* = 1) excluded. SEIFA: Socio-Economic Index For Areas

^*^Denotes statistical significance (*p* < 0.05) compared to the reference category

^a^Chronic health condition includes diabetes, asthma, COPD, CVD, arthritis, osteoporosis, and cancer

^b^Includes anxiety, depression, stress, or any other mental health conditions in the previous 12 months

## Discussion

This paper reports on a community-based population level representative survey of ARI and testing patterns among adults in South Australia with good coverage of population sub-groups by rurality, education level, household income and country of birth. We found that 19.3% of respondents reported ARI symptoms at the time of interview or within the week prior to being interviewed (weekly estimates ranging from 9.9 to 22.7% over the 13 weeks), and that less than a third had sought medical advice for their symptoms. The most common symptoms reported were runny nose and cough. Less than one percent (0.8%) reported fever and cough. These surveys took place during a period of very low COVID-19 activity in the South Australian community where there were 443 confirmed cases (to 30 June 2020), of which 304 were acquired overseas. Therefore, it is assumed that the vast majority of those reporting ARI had illness in the surveys were caused by a non-COVID-19 respiratory pathogen, or from another cause (e.g. allergies). However public health messaging at the time strongly encouraged anyone with even mild respiratory symptoms to seek medical care and be tested for COVID-19. This was also during a period when there were relatively few restrictions on social interaction and mobility in South Australia [18]. In Victoria, levels of ARI were lower [19], and this may be due to higher levels of restrictions impacting on non-COVID-19 respiratory illness in Victoria due to an active outbreak at that time.

In both the univariate and multivariate analysis, older age, larger household size, more chronic conditions, poor or neutral wellbeing, any mental health condition, and psychological distress were risk factors for self-report of ARI symptoms. While some of these factors, such as larger household size, have previously been established as risk factors for ARI, older age is generally protective against ARI [20]. We believe that the association we found may be due to the lack of ability within our study to differentiate chronic from acute ARI symptoms, as well as younger respondents (18–29 years) being underrepresented and those aged 50–69 years overrepresented (unweighted data).

There are relatively few other studies on community prevalence of ARI during the COVID-19 pandemic. In Australia, the only national source of community prevalence for ARI is ‘Flutracker’ [21]; an online community influenza-like illness (ILI) surveillance system. Over the same period as our survey, the Flutracker weekly incidence of self-reported fever and cough was less than 1%, while afebrile illness (with runny nose and sore throat) ARI was between 0.5 to 1.5%; this is considerably lower than the prevalence found in this survey. This difference may be due to varying case definitions, with this survey reporting current symptoms rather than new symptoms. Subsequently, from October 2020, the SAPHS collected information from symptomatic respondents to determine those with acute or chronic symptoms. The proportion of symptomatic respondents reporting acute symptoms (aligning closer to the Flutracker definition) in the SAPHS was 38%, in addition to only having fever and cough (0.8%). To retrospectively apply this proportion over the sample in this study, gives an average of 7.3% with acute ARI symptoms. Those with acute symptoms tended to be younger, and as such the analyses presented here are considered to reflect both those presenting acutely or also with chronic ARI symptoms. The difference between the two surveillance systems could be partly explained by biases generally found in online participatory surveillance systems such as ‘Flutracker’ [22] including over-representation of those in higher socio-economic groups and older ages. These differences emphasise the value of representative surveys to support COVID-19 surveillance and response.

There are significant public health implications to our finding that, within the subgroup with ARI, when asked whether they had had a COVID-19 test, only 18% had already taken a test, with a further 4.3% intending to. COVID-19 testing rates in Australia are high by international standards, and there is strong public health messaging on the importance of getting tested with even minor symptoms. However, our findings indicate that the majority of the population are not being tested when they experience ARI symptoms. The only other relevant Australian data we are aware of comes from Flutracker and shows a similarly low rate of testing uptake. Another Australian study using survey data collected between April to June 2020 has also found that only 49% of people strongly agreed they would get tested if they had COVID-19 symptoms [8]. However, the study participants for this study were recruited online via social media platforms and therefore may not be representative to the general population as compared to our study. This is further borne out by considering absolute testing rates; over the time of the study, South Australia had a crude rate of 1.2 tests per 1000 population per day which is much lower than the community prevalence of ARI found by our survey. Given that this number of tests is likely to include some asymptomatic people, this indicates the relatively low testing uptake amongst those with ARI. Our descriptive and regression analyses suggested lower testing rates in the highest income group ($100,000 and above). It is possible this reflects workers being less likely to present for testing due to the need to self-isolate until test results are returned. However, given how few participants were in the highest income group, the finding should be interpreted cautiously. Population surveys of ARI, such as that presented here, are crucial for public health to monitor, on an ongoing basis, the proportion of symptomatic people being tested. This group should be the main focus of testing, except in the event of an outbreak when testing contacts of cases.

Effective surveillance for COVID-19 requires representative community prevalence data on ARI paired with data on uptake of testing to understand disease patterns and testing rates among different groups. As mentioned above, Flutracker is the only long-term national source of community surveillance for ILI and ARI in Australia and whilst valuable, should be supported by surveys that capture the wider population, along with a range of other demographic, health and wellbeing data. Several syndromic systems in Australia report on ARI presentations [23] however these are all healthcare based, and so inherently biased towards those more likely to seek healthcare. Undertaking regular, representative community-based surveys allows validation of systems such as Flutracker and sentinel surveillance and qualification of potential bias, and in addition, the opportunity to obtain insights into the reasons for testing- related behaviours. As was done in South Australia, adaptation of existing population survey chronic disease surveillance systems is an efficient and effective way to do this.

Like any self-reported system, there is the potential for inherent measurement bias in the reporting of both symptoms and comorbidities. Additionally, our questions on respiratory symptoms did not differentiate between acute versus chronic symptoms. However, we have rectified this in our ongoing ARI syndromic surveillance and commenced collecting this data from October 2020. While we used weighting to account for differences between our sample and target population, we did not collect data on non-respondents. The combined cooperation rate [24] of both surveys was 80.4%. Reporting of both respiratory symptoms and presentation to a health care provider may also have been influenced by the changing context of COVID-19 in the South Australian and broader Australian community during the time period in which these surveys took place. Telephone-based surveys may also under-represent key groups of interest, and this has the potential to bias our results.

## Conclusions

This paper shows the relatively high rates of self-reported ARI found in a usual community setting during a period of very low COVID-19 prevalence, and the relatively low percentage of this group who sought health advice or testing for their symptoms despite consistent public health messaging around this. Ongoing monitoring of testing uptake, including in higher-risk groups, and interventions for understating and improving uptake are key to early detection of disease. Our results demonstrate the feasibility and utility of ongoing community-based representative surveys in addressing these goals.

## Data Availability

The datasets generated and/or analysed during the current study are available from the corresponding author on reasonable request.

## Abbreviations

ARI: Acute Respiratory Infection
CATI: Computer assisted telephone interviewing
CAWI: Computer-assisted web interview
COVID-19: Coronavirus Disease 2019
ILI: Influenza-like Illness
OR: Odds ratios
PHSMS: Population Health Survey Module System
SAPHS: South Australian Population Health Survey
SEIFA: Socio-Economic Index For Areas
